# Effects of Temperature, pH, and NaCl Concentration on Biomass and Bioactive Compound Production by *Synechocystis salina*

**DOI:** 10.3390/life13010187

**Published:** 2023-01-09

**Authors:** Joana Assunção, Helena M. Amaro, Tânia Tavares, F. Xavier Malcata, A. Catarina Guedes

**Affiliations:** 1 CIIMAR-CIMAR-LA—Interdisciplinary Centre of Marine and Environmental Research, University of Porto, Novo Edíficio do Terminal de Cruzeiros do Porto de Leixões, Av. General Norton de Matos, s/n, 4450-208 Matosinhos, Portugal; 2 LEPABE—Laboratory for Process Engineering, Environment, Biotechnology and Energy, Faculty of Engineering, University of Porto, Rua Dr. Roberto Frias, s/n, 4200-465 Porto, Portugal; 3 ALiCE—Associate Laboratory in Chemical Engineering, Faculty of Engineering, University of Porto, Rua Dr. Roberto Frias, 4200-465 Porto, Portugal; 4 FEUP—Department of Chemical Engineering, Faculty of Engineering, University of Porto, Rua Dr. Roberto Frias, s/n, 4200-465 Porto, Portugal

**Keywords:** cyanobacteria, biomass, pigments, antioxidant capacity, Box–Behnken model, optimization

## Abstract

*Synechocystis salina* is a cyanobacterium that has biotechnological potential thanks to its ability to synthesize several bioactive compounds of interest. Therefore, this study aimed to find optimal conditions, in terms of temperature (15–25 °C), pH (6.5–9.5), and NaCl concentration (10–40 g·L^−1^), using as objective functions the productivities of biomass, total carotenoids, total PBPs, phycocyanin (PC), allophycocyanin (APC), phycoerythrin (PE), and antioxidants (AOXs) capacity of *Synechocystis salina* (*S. salina*) strain LEGE 06155, based in factorial design resorting to Box-Behnken. The model predicted higher biomass productivities under a temperature of 25 °C, a pH of 7.5, and low NaCl concentrations (10 g·L^−1^). Maximum productivities in terms of bioactive compounds were attained at lower NaCl concentrations (10 g·L^−1^) (except for PE), with the best temperature and pH in terms of carotenoids and total and individual PBPs ranging from 23–25 °C to 7.5–9.5, respectively. PE was the only pigment for which the best productivity was reached at a lower temperature (15 °C) and pH (6.5) and a higher concentration of NaCl (≈25 g·L^−1^). AOX productivities, determined in both ethanolic and aqueous extracts, were positively influenced by lower temperatures (15–19 °C) and higher salinities (≈15–25 g·L^−1^). However, ethanolic AOXs were better recovered at a higher pH (pH ≈ 9.5), while aqueous AOXs were favored by a pH of 8. The model showed that biomass production can be enhanced by 175% (compared to non-optimized conditions), total carotenoids by 91%, PC by 13%, APC by 50%, PE by 130%, and total PBPs by 39%; for AOX productivities, only water extracts exhibited a (marginal) improvement of 1.4%. This study provided insightful information for the eventual upgrading of *Synechocystis salina* biomass in the biotechnological market.

## 1. Introduction

Cyanobacteria are photosynthetic microorganisms that have been gaining relevance as a potential biotechnological resource due to the window of opportunity associated with environmental-related uses, e.g., bioremediation, CO_2_ mitigation, or even bioenergy production (biodiesel or hydrogen), with them also serving as feed for aquaculture purposes [1,2]. In addition, they are recognized as a potential source of high-value bioproducts, including bioactive compounds [3,4,5]. Phycobiliproteins (PBPs), antioxidants, lipidic components (e.g., carotenoids and polyunsaturated fatty acids (PUFAs)), phenolic compounds, and proteins, among others, are some of the most important explored compounds recovered from cyanobacteria. Those compounds exhibit several pharmacological bioactivities, including antioxidant, antitumor, anti-inflammatory, cardiovascular, and neuro- and hepatoprotective properties [6,7,8]. Due to their non-toxic nature, they also have a potential application as natural food and feed ingredients or colorants (specifically phycobiliproteins and carotenoids in the context of cosmetic formulation, biomedical research, or clinical diagnostics) [4,5,9].

Across the world, the industry has been developing new and innovative products (i.e., food ingredients) from natural sources. To succeed an in such endeavor, while simultaneously supporting competitive and feasible processes, novel (cyanobacterium) strains are to be found and selected, and their culturing optimized, in terms of biomass and metabolite production [10].

Cyanobacteria possess a strong metabolic plasticity, so they can efficiently adapt to their environment, i.e., they regulate metabolite composition in response to biotic variations and abiotic factors. It is well known that some cyanobacterial bioactive compounds (e.g., carotenoids, antioxidants) accumulate under environmental stress (i.e., strong light and/or nutrient deprivation, high salinity), and this triggers adaptive response mechanisms to oxidative stress, with the subsequent acclimatization of cells to the surrounding environment [11]. The modulation of cyanobacteria in response to specific environmental conditions may emerge as a key strategy for the improvement of their growth and/or the production of value-added compounds. To thrive on a commercial scale, a deeper understanding of how cultivation conditions impact on the accumulation of relevant compounds is crucial [11]. Abiotic factors such as light, temperature, nutrient concentration, carbon source, pH, or NaCl concentration have indeed been shown to impact the performance of metabolite synthesis in cyanobacteria; hence, their modulation proves relevant for cyanobacterium growth and metabolite production (and accumulation) [11,12].

Cyanobacterium *S. salina* LEGE 06,155 has been reported to have significant biotechnological potential, not only from an environmental point of view (in terms of bioremediation) but also as a prospective source of bioactive compounds with commercial interest (namely lipids for biodiesel and pigments) [13,14,15,16,17,18]. Therefore, this work aimed to evaluate the influence of temperature, pH, and NaCl concentration to determine optimal conditions using the maximum biomass and bioactive metabolite productivities as objective functions, including the total carotenoids, PBPs [phycocyanin (PC), allophycocyanin (APC), phycoerythrin (PE)], and antioxidant compounds. To attain this goal, a factorial design based on the Box–Behnken model was followed via a three-level run surface response methodology (SRM). This supported the study of the effect of three independent parameters, i.e., temperature (15–25 °C), pH (6.5–9.5), and (initial) NaCl concentration (10–40 g·L^−1^) and their combinations.

## 2. Materials and Methods

### 2.1. Microorganism

*S. salina* LEGE 06,155 was obtained from the Blue Biotechnology and Ecotoxicology Culture Collection (LEGE-CC), CIIMAR (Centre of Marine and Environmental Research of the University of Porto, Portugal) and was kept at 20 °C in BG11 culture medium [19] supplemented with 25 g·L^−1^ of NaCl, with a pH adjusted to 7.2 ± 0.05. Agitation was provided by an airflow of 0.75 L_air_·L^−1^·min^−1^ under a light intensity of 100 µmol_photons_·m^−2^·s^−1^ supplied by fluorescent lamps (Biolux, Osram, Munich, Germany) and a light/dark cycle (L/D) of 16 h/8 h.

### 2.2. Experimental Design

To optimize the productivities in terms of biomass, as well as the total carotenoids, PBPs, and antioxidant compounds, a factorial experiment was performed by applying a three-factor level Box–Behnken design. The temperature (15–25 °C), pH (6.5–9.5), and NaCl concentration (10–40 g·L^−1^) were studied at equidistant levels (−1, 0, 1), as shown in Table 1, for total of 13 runs (in triplicate); the result was plotted using Design-Expert 12 software (Stat-Ease, Minneapolis, MN, USA) [20].

Biomass, total PBPs (PC, APC, and PE), total carotenoids, and antioxidant compound productivities were fitted by a second-order polynomial (quadratic model), and the multiple regression of data was performed to estimate the corresponding coefficients. This could be used to approximate the response and investigate interactions between the factors of temperature, pH, and NaCl concentration ([NaCl]) and to establish the optimum conditions for each parameter. The second-order polynomial was established as follows:

Y = α_0_ + β_1_A + β_2_B + β_3_C + γ1AB + γ_2_AC + γ_3_BC + ⍵_1_A^2^ + ⍵_2_B^2^ + ⍵_3_C^2^ (1)

In which Y is the predicted response; α0 is a constant (intercept); β_1_, β_2_, and β_3_ are linear coefficients; γ_1_, γ_2_, and γ_3_ are two-factor interaction coefficients; ⍵_1_, ⍵_2_, and ⍵_3_ are quadratic coefficients; and A, B, and C are independent variables, i.e., temperature, pH, and [NaCl], respectively.

The polynomial equation was also expressed as surface and contour plots; the fit adequacy of the quadratic model and the associated regression coefficient, r, was determined for every objective function. In addition, the best conditions estimated for each response were scored regarding their desirability. This function varies from 0 to 1, with the highest score representing a completely desirable or ideal response value.

### 2.3. Biomass Production

In all runs, pre-inocula were established over 22 days with BG11 culture medium. To standardize the adaptation of the cyanobacterium to each condition tested, *S. salina* was cultivated as per the conditions associated with the central point of the design, i.e., 25 g·L^−1^ of [NaCl], a pH of 8, and 20 °C. Each run was established with an initial optical density (OD) of 0.1 (OD = λ_680 nm_ − λ_750 nm_), and the experiments were carried out in two L-flat bottom round flasks (1.8 L of working volume) maintained in batch over 22 d. The light was provided by fluorescent lamps (BIOLUX, OSRAM) with an intensity of 150 µmol_photons_·m^−2^·s^−1^ and an L/D cycle of 16 h/8 h. Agitation was provided via continuous air bubbling, with an airflow of 0.75 L_air_·L^−1^·min^−1^. To maintain pH in the medium, different buffer solutions were added to a final concentration of 30 mM MES buffer (VWR Chemicals, Radnor, PA, USA) was used to keep the pH at 6.5, HEPES buffer (PanReach, AppliChem, Chicago, IL, USA) was used to keep the pH at 8, and, finally, CHES buffer (PanReach, AppliChem, Chicago, IL, USA) was used to keep the pH at 9.5. The pH was duly adjusted at the beginning of each experiment, with a tolerance range of ±0.5, and was periodically monitored (on days 0, 1, 4, 6, 8, 11, 13, 15, 18, 20, and 22) with a pH meter (Hanna Instruments, model HI2210, Woonsocket, RI, USA).

### 2.4. Biomass Quantification

The biomass was evaluated by the determination of the OD and dry weight (DW); hence, culture samples were collected regularly (on days 0, 1, 4, 6, 8, 11, 15, 18, 20, and 22). The OD was obtained in a microplate spectrophotometer reader (Multiscan Go, ThermoFisher, Waltham, MA, USA) at a wavelength of λ = 680–750 nm for each biological replicate and assayed in triplicate.

The DW was obtained by filtering aliquots of culture through preconditioned 0.45 µm (pore size) glass microfiber filter paper (Whatman GF/C, Maidstone, UK), with them being dried further at 100 °C until a constant weight was achieved. To avoid the salt effect and to standardize the DW conditions, each sample, in particular those with an [NaCl] of 25 and 40 g·L^−1^, was diluted up to 10 g·L^−1^ NaCl, as described elsewhere [21]. All DW tests were performed in duplicate.

The maximum biomass productivity (P_x_, mg·L^−1^·d^−1^) was determined using DW experimental data and estimated based on Px (t) = (X_max_ − X_0_)/(t − t_0_), where X_0_ is the biomass at the beginning of the experiment (time t_0_) and X_max_ denotes the maximum biomass concentration attained at time t.

### 2.5. Pigment Extraction and Quantification

#### 2.5.1. Total Carotenoids and Phycobiliprotein Extraction and Quantification

For the total carotenoids and PBPs, a sequential extraction was performed with ethanol (96%, *v*/*v*) and water, respectively, on days 0, 1, 4, 6, 8, 11, 15, 18, 20, and 22. These extracts were also used to ascertain the antioxidant capacity, as described in Section 2.6.

To analyze the total carotenoid content, 3 mL aliquots of different culture conditions were harvested and centrifuged (2744× *g* for 10 min); the supernatant was discarded, and 3 mL of ethanol was added to the pellet with glass beads. Each sample was extracted in a *Precellys* homogenizer (Bertin Technologies, Montigny-le-Bretonneux, France), using a 6 min cycle at 8000 rpm (30 s homogenization with 40 s stopping intervals). Ethanolic extracts were centrifuged at 2744× *g* for 10 min, kept under dimmed light, and stored at −4 °C until pigment content and antioxidant capacity determination.

The total carotenoid content was determined spectrophotometrically (Shimadzu UV-1800, Kyoto, Japan) according to Lichtenthaler and Buschmann (2001) [22]. The absorption was read at λ = 470, λ = 664, and λ = 648 nm, and the content was calculated as follows:

Chl *a* (μg·mL^−1^) = (13.36 A_664_) − (5.19 A_648_) (2)

Total carotenoids (μg·mL^−1^) = (1000 A_470_) − (2.13 Chl *a*)/209 (3)

For the extraction of total PBPs, the remaining pellet of ethanolic extraction was added with 3 mL of water, and then samples were subjected to vortexing for 20 s. The extract was centrifuged at 2744× *g* for 8 min and then kept under dark conditions before the analysis for pigment content and antioxidant capacity.

The determination of the PBP content, namely phycocyanin (PC), allophycocyanin (APC), and phycoerythrin (PE), was via the spectrophotometric measurement of absorbance at λ = 562, 615, and 652 nm, followed by the application of Bennett and Bogorad′s equations [23], see Equations (4)–(6):

(4) $$\mathrm{PC}\;(\mathrm{mg}\cdot {\mathrm{mL}}^{-1})=(A615)\;-\;0.474\;A652)/5.34$$

(5) $$\mathrm{APC}\;(\mathrm{mg}\cdot {\mathrm{mL}}^{-1})=[(A652)\;-\;(0.208\;A615)]/5.09$$

(6) $$\mathrm{PE}\;(\mathrm{mg}\cdot {\mathrm{mL}}^{-1})=[(A562)\;-\;2.41(\mathrm{PC})\;-0.849\;\left(\mathrm{APC}\right)]/9.62$$

The PBP productivities were expressed in mg·L^−1^·d^−1^.

#### 2.5.2. Determination of Total Carotenoids Profile and Quantification

For the run that achieved the highest total carotenoid productivities, the profile and content in carotenoids and chl *a* were duly determined.

Regarding carotenoid HPLC analysis, 10 mL of biomass was first harvested and centrifuged at 2744× *g* for 10 min. A volume of 5 mL of acetone (100%) and 100 µL of the internal standard solution of trans-β-Apo-8′-carotenal (170 mg·L^−1^; ExtraSynthase ≥ 98.0% (UV)] was added to the pellet to control the process of extraction and carotenoid quantification. Samples were accordingly subjected to *Precellys* homogenization at 8000 rpm for 6 min and then centrifuged (2744× *g* for 10 min); the supernatant was kept at −20 °C, until further analysis.

The identification and quantification of carotenoids by HPLC were performed as described elsewhere [24]. Acetonic extracts were evaporated in a vacuum rotary evaporator and resuspended in 400 µL of acetone:ethyl acetate (9:1) previously filtered through a PTFE filter syringe (membrane solutions, 0.22 µm) before injection. HPLC (Waters Alliance 2695, Milford, MA, USA) was equipped with a photodiode array (PDA) detector, an FLR detector, and a column heater to maintain a constant temperature throughout the analysis. The stationary phase was constituted by a LiChroCART^®^ 250-4 C18-reversed-phase column (250 × 4 mm, 5-μm bondapack) (Merck, Darmstadt, Germany), while the mobile phase was ethyl acetate (solvent A) and 9:1 (*v*/*v*) acetonitrile:water (solvent B). The overall flow rate was set at 1 mL·min^−1^, with a column pressure of 3000 bar, a constant temperature of 25 ± 2 °C, and a gradient regarding solvents A and B over time as follows: 0–31 min (0–60% A); 31–36 min (60% A); 36–38 min (60–100% A); 38–43 min (100% A); 43–50 min (100–0% A); and 50–55 min (0% A). Spectral data from all peaks were collected within the range of 250–750 nm.

The carotenoids and chl *a* were identified via UV-visible photodiode spectra and comparison of the retention times (RT) with those of standard zeaxanthin (Extrasynthese, ≥98.0% (UV) with an RT of 13.2 min, chl *a* (Sigma, ≥96.0% (UV)) with an RT of 24.6 min, echinenone with an RT of 25.2 min, and β-carotene (Extrasynthese, ≥98.0% (UV)) with an RT of 32.8 min, performed with the aid of calibration curves prepared with such standards. Three biological replicates were analyzed, and the results were expressed in µg·g_DW_^−1^. Total carotenoid productivities were expressed in mg·L^−1^·d^−1^.

### 2.6. Determination of Antioxidant Capacity

To assess the total antioxidant capacity of ethanolic and aqueous extracts, the ABTS^•+^ assay was performed using a microplate spectrophotometer reader (Multiscan Go, ThermoFisher, Waltham, MA, USA) according to Guedes et al. (2013) [25] as adapted for spectrophotometer plates by Granados-Guzman et al. (2017) [23]. Briefly, ABTS radical cation was produced by reacting potassium persulfate (Sigma-Aldrich, St. Louis, MO, USA) (0.66 mg·mL^−1^) and ABTS (Sigma-Aldrich, St. Louis, MO, USA) (3.84 mg·mL^−1^). For the assay, 63 µL of extract sample was added to 180 µL of ABTS solution, so that the final absorption ranged from 0.68–0.72. All samples were incubated over 6 min, and the absorbance was read in triplicate at λ = 734 nm.

For all assays, the percent inhibition was calculated by Equation (7):

% inhibition = [(Abs_sample_ − Abs_blanck sample_) − Abs_control_]/Abs_control_ × 100% (7)

where Abs_sample_ denotes the extract absorbance, Abs_blanck_ the solvent absorbance of ABTS reagent, and Abs_control_ the absorbance of ethanol or water. The results were plotted based on two calibration curves that were previously established with Trolox dissolved in ethanol and water, respectively. The antioxidant capacity was expressed in mg Trolox equivalents (TE) per liter per day (mg_TE_·L^−1^·d^−1^).

### 2.7. Statistical Analysis

The analysis of variance (ANOVA) utilized the Design-Expert 12.0 program (Stat-Ease, Inc., Minneapolis, MN, USA and was used to estimate the significance levels for each assessed parameter. By performing a lack of fit test, the results of the model and the observed results were duly compared. The model was validated when the statistical significance was higher than 0.05.

The statistics for the carotenoid content, identified by HPLC, were performed via GraphPad Prism (version 8) (GraphPad Software, San Diego, CA, USA), using a one-way analysis of variance (ANOVA) with Tukey′s multiple comparison test. A *p*-value < 0.05 was regarded as significant.

## 3. Results

### 3.1. Experimental Design

A total of 13 experimental runs (*n* = 3) were performed following the Box–Behnken design to find the optimal conditions in terms of temperature, pH, and [NaCl] for productivity regarding biomass, pigments (total PBPs, PC, APC, PE, and total carotenoids), and antioxidant capacity. For all parameters analyzed, the chosen model was statistically significant, with a *p*-value < 0.001; the underlying equations for the predicted responses are shown in Table 2. The regression model exhibited a good fitness: the R^2^ ranged from 0.72–0.98 (Table 2), thus indicating a reasonably high degree of correlation between the experimental and predicted values.

#### 3.1.1. Biomass Productivity

**Figure 1:**
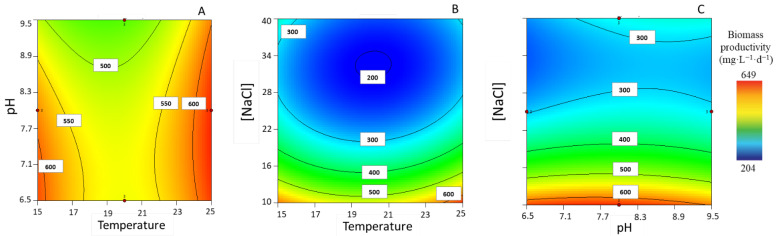
Response surface plots for highest biomass productivity (mg·L^−1^·d^−1^), with corresponding interaction effects of the parameters of temperature, pH, and [NaCl], after setting the (**A**) [NaCl] to 10 g·L^−1^, (**B**) pH to 7.5, and (**C**) T to 25 °C.

The maximum biomass productivity for each experimental condition was determined. The experimental values for biomass productivities ranged from 212.47 ± 3.46 to 641.59 ± 5.57 mg_DW_·L^−1^·d^−1^. Regarding impact factors on biomass productivity, it would appear that the effect of high temperatures with intermediate levels of pH (i.e., 8) favors biomass; however, it can also be observed that biomass productivity is likely to increase at lower temperatures, when combined with a low pH (i.e., 6.5) (Figure 1A). On the other hand, the combined effect of a low [NaCl] and a high temperature leads to an increase in biomass productivity (Figure 1B), and the same is observed for intermediate levels of pH (Figure 1C). The model also showed that [NaCl] by itself has a strong influence on biomass productivity; the higher the [NaCl], the lower the biomass productivities (data not shown).

#### 3.1.2. Total Carotenoid Productivity

**Figure 2:**
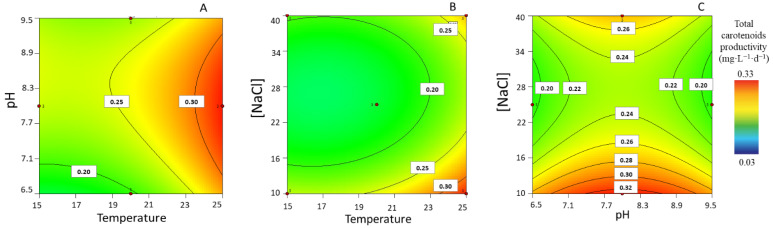
Response surface plots for highest total carotenoid productivity (mg·L^−1^·d^−1^), with corresponding interaction effects of the parameters of temperature, pH, and [NaCl], after setting the (**A**) [NaCl] to 10 g·L^−1^, (**B**) pH to 8, and (**C**) T to 25 °C.

The maximum productivity regarding total carotenoids varied between 0.03 ± 0.00 and 0.33 ± 0.00 mg·L^−1^·d^−1^. The surface response graphs showed that intermediate pH levels and a higher temperature positively influence the productivity of carotenoids (Figure 2A). Moreover, when pH levels are set to 8, a higher temperature combined with a low [NaCl] would appear to favor the accumulation of carotenoids (Figure 2B). The same holds true for a temperature set at 25 °C, with the total carotenoid productivity maximized at an intermediate pH and lower [NaCl] (see Figure 2C).

#### 3.1.3. Total PBPs

The maximum productivity of total PBP ranged from 0.99 ± 0.02 to 2.96 ± 0.04 mg L^−1^·d^−1^.

**Figure 3:**
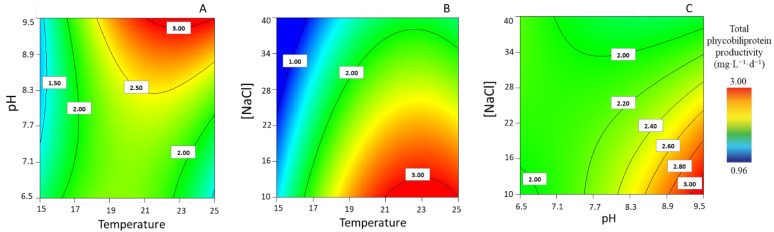
Response surface plots for highest total phycobiliprotein productivity (mg·L^−1^·d^−1^), with corresponding interaction effects of the parameters of temperature, pH, and [NaCl], after setting the (**A**) [NaCl] to 10.9 g·L^−1^, (**B**) pH to 9.48, and (**C**) T to 22.9 °C.

Response surface plots showed that a low [NaCl] (10.9 g·L^−1^) and the combination of a high pH and high temperature (≈23 °C) contribute to increasing PBP productivity (Figure 3A). In addition, the combination of a low [NaCl] and higher temperatures at a high pH results in an enhancement of the total PBP productivity (Figure 3B); when the temperature was ≈23 °C, both a low [NaCl] and a high pH appeared to improve the total PBP productivity.

#### 3.1.4. PC Productivity

**Figure 4:**
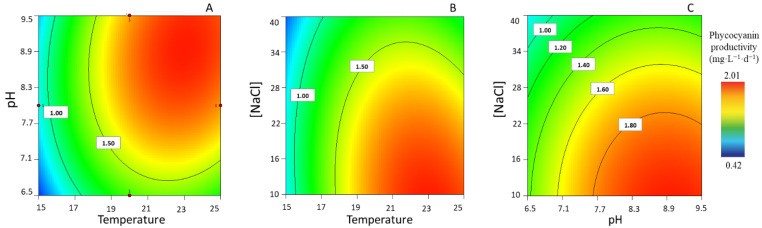
Response surface plots for highest phycocyanin productivity (mg·L^−1^·d^−1^), with corresponding interaction effects of the parameters of temperature, pH, and [NaCl], after setting the (**A**) [NaCl] to 10 g·L^−1^, (**B**) pH to 8.96, and (**C**) T to 22.9 °C.

According to our experimental values, maximum PC productivities varied between 0.42 ± 0.00 and 1.99 ± 0.02 mg·L^−1^·d^−1^. The relationship between the pH and temperature (at low [NaCl] of 10 g·L^−1^) was observed to have a significant impact on PC productivity (Figure 4A). In addition, the relationship between temperature and [NaCl] is relevant at a pH ≈ 9, thus impacting positively upon the maximum production of PC (Figure 4B). Lower productivities are predicted when the [NaCl] increases simultaneously with low pH levels in addition to low temperatures (Figure 4C).

#### 3.1.5. APC Productivity

**Figure 5:**
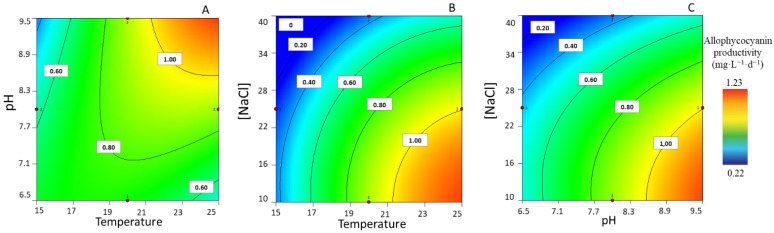
Response surface plots for highest allophycocyanin productivity (mg·L^−1^·d^−1^), with corresponding combined effects of the parameters of temperature, pH, and [NaCl], after setting the (**A**) [NaCl] to 10 g·L^−1^, (**B**) pH to 9.5, and (**C**) T to 25 °C.

Focusing on APC maximum productivity, the values produced ranged from 0.24 ± 0.01 to 1.23 ± 0.06 mg·L^−1^·d^−1^. These results also indicated that at low levels of [NaCl] (i.e., 10 g·L^−1^), the combination of a high pH (9.5) and a temperature (25 °C) impacts positively upon APC productivity (Figure 5A). When setting a high pH (i.e., a pH of 9.5), the combined effect of a low [NaCl] and a higher temperature potentiates high APC productivities (Figure 5B); likewise, at 25 °C, the combined effect of low concentrations of NaCl and a more alkaline pH (9.5) led to higher maximum APC productivities (Figure 5C).

#### 3.1.6. PE Productivity

**Figure 6:**
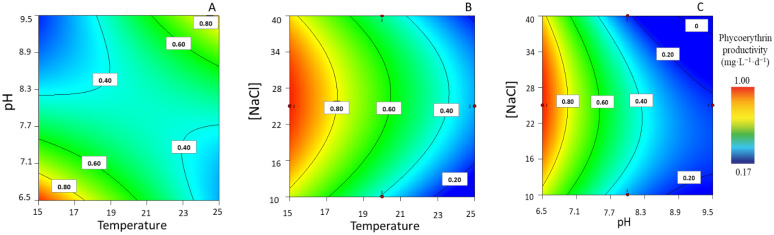
Response surface plots for highest phycoerythrin productivity ((mg·L^−1^·d^−1^), with corresponding interaction effects of the parameters of temperature, pH, and [NaCl], after setting the (**A**) [NaCl] to 26.3 g·L^−1^, (**B**) pH to 6.5, and (**C**) T to 15 °C.

Regarding PE maximum productivities, experimental values indicated a variation between 0.17 ± 0.00 and 0.96 ± 0.03 mg·L^−1^·d^−1^. A closer look at Figure 6 suggests that, when [NaCl] is set at intermediate levels (i.e., ≈26 g·L^−1^), temperature and pH exhibit interaction effects in opposite directions (Figure 6A). This means that PE can be produced under a low temperature and pH and that there is an antagonist interaction effect under a higher temperature (25 °C) and pH (9.5); however, according to the model, the maximum PE productivity appears to be associated with a low temperature and moderate [NaCl]. Despite being less significant, the interaction between [NaCl] and temperature was observed under low pH values (pH = 6.5) and contributes to high PE productivities (Figure 6B). A low pH and intermediate [NaCl] exert a positive effect upon PE productivity under low temperatures (Figure 6C).

#### 3.1.7. Ethanolic Antioxidant Compound Productivity

**Figure 7:**
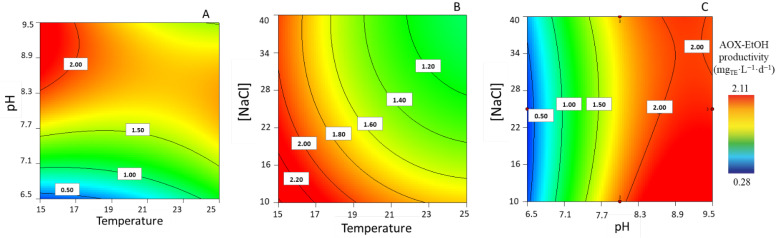
Response surface plots for highest antioxidant capacity of ethanolic extracts productivity (mg_TE_.L^−1^·d^−1^), with corresponding combination effects regarding the parameters of temperature, pH, and [NaCl], after setting the (**A**) [NaCl] to 16.28 g·L^−1^, (**B**) pH to 9.45, and (**C**) T to 15 °C.

Regarding the antioxidant compound maximum productivity in ethanolic extracts, experimental data varied between 0.28 ± 0.00 and 2.10 ± 0.01 mg_TE_·L^−1^·d^−1^. The surface response encompassing the different parameters, in terms of AOX-EtOH productivities, is shown in Figure 7. After setting a lower [NaCl] (i.e., 16 g·L^−1^), the combination of a lower temperature and higher pH leads to higher productivities (Figure 7A). The plot in Figure 7B shows that, for a pH set at higher values (i.e., 9.5), the combination of a low temperature and a low [NaCl] favors AOX productivities. Furthermore, the plot in Figure 7C would appear to support the notion that a low temperature (15 °C) yields a higher AOX productivity, irrespective of [NaCl], as long as the pH is high (i.e., 9.45).

#### 3.1.8. Aqueous Antioxidant Compounds Productivity

**Figure 8:**
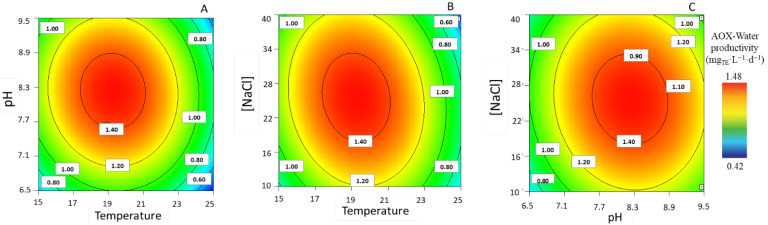
Response surface plots for highest antioxidant capacity of water extracts (mg_TE_·L^−1^·d^−1^), with corresponding combination effects of the parameters of temperature, pH, and [NaCl], after setting the (**A**) [NaCl] to 25.5 g·L^−1^, (**B**) pH to 8.2, and (**C**) T to 19 °C.

Regarding the maximum productivity of antioxidant compounds in successive aqueous extracts, the experimental values ranged from 0.43 ± 0.01 to 1.45 ± 0.02 mg_TE_·L^−1^·d^−1^. With the NaCl concentration at intermediate values (i.e., 25 g·L^−1^), the interaction between temperature and pH is observed at intermediate levels and results in a higher productivity concerning AOX-water compounds (Figure 8A). Similarly, when the pH is set at 8.3, intermediate temperatures and [NaCl] levels (i.e., 19 °C and 25 g·L^−1^) are better in terms AOX-water compound productivity, even though no true local maximum is found (Figure 8B,C). At a temperature of 19 °C, it is possible to observe a combination effect in terms of [NaCl] and pH, again at intermediate levels, with respect to water-soluble AOX compounds.

### 3.2. Model Optimal Conditions

For each parameter evaluated, the optimal conditions to attain maximum productivity were investigated (Table 3). In addition, the maximum values for productivity as predicted by the model were compared to that under non-optimized conditions, namely, experimental values generated at the central point (T = 20 °C, pH = 8, and [NaCl] = 25 g·L^−1^) (Table 3).

The optimal conditions found for biomass productivity were T = 25 °C, pH = 7.5, and [NaCl] = 10 g·L^−1^, with a maximum predicted productivity of 637.43 ± 20.56 mg·L^−1^·d^−1^ (desirability = 0.974). When compared to the non-optimized conditions, the maximum predicted value represented an increase of ≈175% (see Table 3).

The pattern for the best conditions to achieve higher productivities in terms of total carotenoids were similar, i.e., T = 25 °C, pH = 8, and [NaCl] = 10 g·L^−1^, with a maximum of 0.33 ± 0.04 mg·L^−1^·d^−1^ (desirability = 0.992), as estimated by the model. Compared to the conditions prevailing at the central point, the total carotenoid productivity increased by ≈90.7% (see Table 3).

To obtain higher productivities in terms of total PBP, the model pointed towards T ≈ 23 °C, pH ≈ 9.5, and [NaCl] ≈ 10 g·L^−1^, yielding a maximum predicted value of 3.09 ± 0.32 mg·L^−1^·d^−1^ (desirability = 1), which entails a 39% increase in total PBP productivity (Table 3) when compared to experimental values taken under intermediate conditions.

In terms of PC, a combination of T = 23 °C, pH ≈ 9, and [NaCl] = 10 g·L^−1^ proved the best conditions for productivity, with an estimated maximum of 1.97 ± 0.20 mg·L^−1^·d^−1^ (desirability = 0.974). A comparison with the central value resulted in an increase of ca. 19% in this case (see Table 3).

The model indicated that the best conditions to reach high APC productivities are T = 25 °C, pH = 9.5, and [NaCl] = 10 g·L^−1^, with a maximum estimated value of 1.17 ± 0.12 (desirability = 0.940). A comparison between the experimental results taken at the central value and the predicted value resulted an increase in APC maximum productivity by 50% (see Table 3).

The best conditions determined to achieve higher PE productivities require lower temperatures and a lower pH combined with a slightly higher [NaCl] than in the case of PC and APC; in this case, T = 15 °C, pH = 6.5, and [NaCl] ≈ 25 g·L^−1^, with maximum predicted of 0.99 ± 0.09 (desirability = 0.968). Compared to the non-optimized conditions, the predicted response increased by 130%.

Concerning the best conditions to increase the productivity of AOX compounds in ethanolic extracts, the model provided the following: T = 15 °C, pH ≈ 9.5, and [NaCl] ≈ 15%, with a predicted maximum of 2.24 ± 0.18 mg_TE_·L^−1^·d^−1^ (desirability = 1). Compared to the intermediate experimental point, the maximum productivity of EtOH-soluble AOX compounds corresponds to a 6%-increase.

According to the model, the best conditions for an increase in productivity of AOX compounds in water extracts are T = 19.2 °C, pH = 8.2, and [NaCl] = 25.5 g·L^−1^, with a predicted value of 1.47 ± 0.09 mg_TE_·L^−1^·d^−1^ (desirability = 0.988) (see Table 3). The improvement was marginal in this case, ca. 1.381% (see Table 3).

### 3.3. HPLC Carotenoid Profile

The selected model indicated that the best conditions for *S. salina* to attain higher productivity in terms of carotenoids essentially coincide with those prevailing during one of the runs (T = 25 °C, pH = 8, and [NaCl] = 10 g·L^−1^). Hence, the carotenoid profile and content (Table 4) of *S. salina* were determined. Chl *a* was found at a concentration of up to 86% of the total pigments identified by HPLC, whilst carotenoids represented only 14%. Nevertheless, 90% of the total carotenoids (*p* < 0.05) were β-carotene, zeaxanthin, and the unidentified carotenoid (peak 1, Table 4). Echinenone was found in significantly minor concentrations (3.54% of the whole inventory of carotenoids) (*p* < 0.05), as were other unidentified carotenoids (6.88%) (see Table 3).

## 4. Discussion

This study provided an insight into the best conditions in terms of temperature, pH, and [NaCl] associated with the highest productivities of several compounds of interest within the biotechnological field (i.e., food and nutraceuticals), namely, total carotenoids, total PBPs, and antioxidant compounds in ethanolic and aqueous extracts.

The conditions found for each objective function are different from each other. It is well recognized that the best conditions to attain high productivities in terms of biomass do not necessarily coincide with the best conditions to achieve the highest productivities in terms of pigments or antioxidant compounds [26].

### 4.1. Biomass Production

Temperature, pH, and [NaCl] influence several metabolic activities, including the function of intracellular enzymes, nutrient uptake, photosynthesis (e.g., rate and extent of photosynthetic electron chain transport), or membrane stability, and ultimately growth and maximum biomass accumulation [27]. However, *S. salina* was seen to efficiently adapt to physicochemical variations imposed upon the culture, as its growth was not inhibited within the studied ranges. The combination of a high temperature (25 °C), neutral levels of pH (7.5), and lower [NaCl] (10 g·L^−1^) appeared to favor biomass maximum productivity.

The optimum temperature for growth and biomass production are variable; in the case of *S. salina*, 25 °C maximized biomass productivity. In fact, for other strains of *Synechocystis* (i.e., strain 6803), 30 °C and a pH of 7–8 were recommended for their cultivation [28]. To define suitable ranges for the optimization of *S. salina* productivities, a preliminary study was conducted in which our strain was subjected to 30 °C (under a higher [NaCl] of 40 g·L^−1^ and a pH of 8); however, its growth was compromised and the culture collapsed (data not shown). It is worth mentioning that Box–Behnken is a model that has to be applied at equidistant levels for the studied factors. This means that, despite observing interesting results at 15º C (in preliminary tests, data not shown), we decided to go for 20 °C because it is a mild temperature. Then, accordingly, 25 °C was the chosen temperature for the model. Further temperatures were not tested close to 30 °C (e.g., 28 °C) because similar results in terms of collapse could occur. Additionally, from an industrial point of view, temperatures above 25 °C can have higher costs at production level, which could be economically unfeasible.

Usually, the optimum growth rate is achieved by increasing the temperature to its optimum level; this is a strain-dependent factor because the range of growth temperatures and the optimal growth temperature depend on the microorganism at stake [6]. For instance, Nalley et al. (2018) found that *Anabaena cylindrica* can grew between 9 and 33 °C, but its optimal temperature was established at 31 °C under an irradiance of 100 μmol_photons_·m^−2^·s^−1^.

*Arthrospira platensis* and *Arthrospira fusiformis* underwent, in turn, a drop in their accumulated biomass when subjected to low (15 °C) and high (40 °C) temperatures, but with maximum biomass productivity at 32 °C (up to 2.4 g·L^−1^) and 37 °C (up to 2.3 g·L^−1^), respectively. Rapid shifts in temperature may redirect metabolic processes and thus change the biochemical composition of cells. This will create high stress; if the capacity to acclimate to new conditions is limited, growth and biomass production may cease, causing, in extreme cases, the culture tent to collapse through the inhibition of its photosynthetic apparatus [29].

*Synechocystis salina* is a marine cyanobacterium. As this species was usually maintained in our collection under a NaCl concentration of 25 g·L^−1^, it was decided to observe concentrations close to the levels of marine water (40 g·L^−1^). A high [NaCl] significantly reduced the maximum biomass productivities (*p* < 0.05) of *S. salina*, even when compared to the mid-point conditions (i.e., [NaCl] = 25 g·L^−1^). However, the results suggest that this strain can adapt to high [NaCl] because the cultures did not collapse. High concentrations of Na^+^ and Cl^-^ in the medium have been claimed to destabilize ion cell homeostasis and consequently lead to osmotic stress, with severe repercussions regarding growth and biomass accumulation [30,31]. For instance, *Thermosynechococcus* sp. CL-1 used in a CO_2_ fixation process and subjected to [NaCl] of 8, 18, and 29 g·L^−1^, decreased its biomass productivities from 90.31 to 49.58 and 6.46 mg·L^−1^·h^−1^, respectively [30]. This was a consequence of a decrease in photosynthetic efficiency (i.e., the inhibition of the electron transport chain) but also the redirection of energy for the active pumping of Na^+^ ions and the production of carbohydrates as salt protectors to counterbalance the cell stress [32]. Ion imbalance is also prone to affect the photosystem-II center reaction, with an alteration in the water oxidation complex and the production of reactive oxygen species (ROS) [27,33].

The results showed that low NaCl concentrations (10 g·L^−1^) favored *S. salina* biomass production, which is of interest from a commercial point of view.

### 4.2. Total Carotenoids and Profile

Regarding total carotenoids, the model showed that maximum productivities are obtained under higher temperatures (25 °C), an intermediate pH (8) and lower [NaCl] (10 g·L^−1^). In general, the increase in carotenoid synthesis in culture occurs under extreme conditions, particularly in terms of light intensity, but also under extreme salinity (high [NaCl]) or pH [11,34]. This is particularly true for microalgae; however, cyanobacteria such as *Synechoccystis* possess a higher ability for acclimatization, so the response in terms of carotenoid production cannot be so linear. Some of the most common carotenoids produced by cyanobacteria include β-carotene and zeaxanthin, the ketocarotenoid echinenone, and the monocyclic glycoside myxoxantophyll [35]. However, their carotenoid profiles are moderately variable among genera and even among strains [36]. In cyanobacteria, carotenoids are usually found in photosynthetic membranes, where they are synthesized. These pigments are assumed to play a (minimal) role in light harvesting in cyanobacteria, yet they protect against ROS and photo-oxidative stress despite their important structural stability, conveyed by pigment-protein complexes to the photosynthetic apparatus [37,38,39]. Despite optimized maximum carotenoid productivities not corresponding to more extreme conditions in terms of cultivation, this is not the case with temperature, which could play a major role in these results. For instance, Kłodawska et al. (2019) [40] showed that sub-optimal temperatures (15° and 37 °C) led to the lowest total carotenoid content in *Anabaena* sp. PCC 7120, whilst temperatures of 23–30 °C were best to increase the total carotenoid content; however, their composition varied, because 23 °C was the best temperature to produce β-carotene, whilst 30 °C led to a higher accumulation of ketocarotenoids (i.e., echinenone, canthaxanthin, and keto-myxoxanthophyll). Interestingly, the results of this agree with Ismaiel et al. (2016) [41], who studied the effect of pH (7.5–10) on carotenoid production by *Arthrospira platensis* and reported a high content at a pH of 8 and 9 (with no statistical differences).

A higher [NaCl] did not trigger a response in terms of carotenoid productivity, as would be expected from studies elsewhere [30,31]. The authors of those studies observed the up-regulation/production of carotenoid protective pigments (i.e., β-carotene and zeaxanthin) when high salinities were attained. However, other studies encompassing different species corroborate the poor accumulation of total carotenoids under high-salt concentrations [21,31]. For instance, Pagels et al. (2020) [21] optimized salinity in terms of NaCl concentration (10 to 30 g·L^−1^) together with temperature (20–30 °C) and pH (6–9). The optimal concentration was set at 10 g·L^−1^, combined with a pH of 9, a temperature of 20 °C, and a carotenoid productivity of 2.04 mg·L^−1^·d^−1^. Regarding the three studied factors, the NaCl concentration had the least impact in terms of carotenoid production. In another study, *Synechocystis* sp. CCNM 250 was tested for different concentrations of NaCl (0.2 M, 0.4 M, 0.6 M, 0.8 M, and 1 M), and reached a higher carotenoid content (7.05 mg·g_DW_^−1^) under a low [NaCl] (0.2 M ≈ 10 g·L^−1^) [31].

The carotenoid profile of *S. salina* was established under the best conditions predicted by the model. Its profile was unveiled and was similar to that reported by Assunção et al. (2021) [18] in terms of ethanolic extracts (single extraction) including zeaxanthin, echinenone, and β-carotene but not α-carotene, β-carotene-5,6-epoxide, and lutein. Despite the solvent for extraction being the same, the biomass and volume of solvent used were different, and the carotenoid concentration and composition also changed based on distinct culture conditions including irradiance, temperature, pH, and [NaCl] [34,42]. In the case of Assunção et al. (2021) [18], their study was performed under a similar temperature (25 °C) but with a light irradiance of 100 μg_photons·_m^−2^·s^−1^, a pH of 7.2, and a [NaCl] of 25 g·L^−1^, using Z8 for culture medium.

### 4.3. Phycobiliproteins

PBPs are water-soluble pigments, with crucial relevance in the photosynthetic apparatus of cyanobacteria. PBPs play a major role in the light-harvesting complex via funneling energy through chl *a* and photosynthetic reaction centers [9,43]. One consequence of *S. salina* growth conditions is the changes in pigment composition in response to acclimatization, with these becoming more photosynthetically efficient [21]. This can explain why the PBP maximum productivities were different when affected by temperature, pH, and [NaCl]. In the case of total PBPs and PC, the conditions to achieve maximum productivities were very similar in terms of temperature (23 °C) and [NaCl] (10 g·L^−1^), but note with respect to pH, which was higher for total PBPs (pH = 9.5). Despite this difference, those results were expected since PC can account ca. 60–75% of the total PBPs [18]. In this study, PC corresponded to 66% of the total PBP content under the best conditions of production.

For high productivities of APC, a lower [NaCl] (=10 g·L^−1^) was predicted, concomitant with a higher temperature (i.e., 25 °C) and pH (i.e., 9.5). On the other hand, when compared to individual PBPs, the conditions to reach PE maximum productivity deviate not only in terms of temperature and pH, i.e., a low temperature (15 °C) and low pH (=6.5), but also because this was the only pigment that appeared to favor a higher [NaCl] (i.e., ≈25 g·L^−1^).

The temperature has been reported to be an important factor, affecting the total PBP production; however, their relative composition can change with other culture parameters (i.e., light conditions, nitrogen, and carbon availability), with this being strongly species-dependent. For instance, an optimum temperature of 30 °C was reported for *Anabaena* NCCU-9 in the context of total PBP production (≈127.02 mg·g_DW_^−1^), with a decrease at 20 °C (23.6%) and 40 °C (38%) [44]. In *Plectonema boryanum* UTEX 485, the PBP content was significantly reduced at 15 °C when compared to 29 °C (under a light intensity of 150 μmol_photons_·m^−2^·s^−1^). PBP productivities can be reduced at low or high temperatures [45]; temperatures above the optimum likely inhibit regular cell metabolic activities and may even promote protein denaturation in extreme cases, which negatively impacts growth and productivity. Conversely, low temperatures reduce cellular metabolic activities, hampering the synthesis of the building blocks necessary for the synthesis of PBPs. Several studies [45,46] have experimented with the combination of light intensity and temperature with the aim of assessing PBP production in different cyanobacteria; as for any other pigment, the combined effect with light will always prove a relevant factor that deeply affects PBPs’ productivities [6].

The medium pH also affected PBP synthesis; the maximum productivities of *S. salina* were enhanced by a more alkaline pH (≈9–9.5), except for PE. A pH of 7–9 is apparently optimum for PBP accumulation, depending on the species at stake [44,47]. For instance, Pagels et al. (2020) [21] found a pH of 9 to be optimum (under 20 °C and [NaCl] = 10 g·L^−1^) to attain maximum total PBP productivities (4.14 ± 0.71 mg·L_culture_^−1^·d^−1^) in *Cyanobium* sp. According to Maurya et al. (2014) [48], a pH of 8 was found to be optimal for total PBPs in *Gloeocapsa* sp., *Lyngbya* sp., *Synechocystis* sp., and *Anabaena* sp. Hong and Lee (2008) [49] showed that a pH of 7.5 was optimum for growth and higher PBP production by *Synechocystis* (strain sp. PCC 6701) under 25 °C and a light intensity of 25 μmol_photons_·m^−2^·s^−1^.

Interestingly, maximum PE production was favored under a considerably more acidic pH (6.5). This can be explained by the different responses of *S. salina* to variations in environmental conditions, which lead to shifts in metabolism, and responses to acclimatization that can favor one compound over another in order to maintain cell homeostasis. Keithellakpam et al. (2015) [47] stated that, for certain strains of *Phormidium* sp. and *Nostoc* sp. PE, accumulation appears to be favored under a pH between 6 and 7. An extreme pH or high shifts in pH may lead to an internal electrostatic attraction that alters the charge of proteins toward a net positive charge, which compromises biochemical and photosynthetic functions in cyanobacterial cultures, meaning that PBPs and the phycobilisome will undergo decay owing to protein denaturation.

The [NaCl] exhibited an important role in PBP productivity. According to Lee et al. (2021) [50] increasing the [NaCl] in the culture medium may have an elicitation effect on PBP content (i.e., APC and PC); they investigated the effects of various NaCl concentrations (0, 0.4, 0.8, and 1.2 M) in photosynthetic pigments, and the highest production of APC (4.08 mg·L−^1^) and PE (1.70 mg·L−^1^) were achieved under 1.2 M NaCl conditions. However, for this study only seemed legitimate for PE pigment. López-Pacheco et al. (2020) [51] subjected *Lyngbya purpurem* to different salinities (12.5, 25 and 50 g·L^−1^) and observed an increase in PC concentration up to 40.4 ± 2.23 mg·g_DW_^−1^ under a medium salinity concentration (25 g·L^−1^). In another study, concentrations equal or above 0.8 M (≈47 g·L^−1^) were found to reduce the PBP concentration [52]. On the other hand, several studies correlated lower salinity contents (i.e., 0.01 M ≈ 0.058 g·L^−1^) to an increased production in terms of PBPs [26,53]. An extreme (high) [NaCl] was reported to produce a quick intake of sodium ions, thus resulting in the detachment of phycobilisome from thylakoid membranes, with a subsequent reduction in the photosynthetic rate and uptake [6].

### 4.4. Antioxidant Capacity Extracts

Cyanobacteria can easily adapt to the most extreme environmental stresses in Nature because their complex metabolism is susceptible to adjustment as a protective strategy against abiotic stresses (UV radiation, osmotic stress, extreme temperature, or pH). One of these strategies is the production of secondary metabolites, such as antioxidants, phenolic compounds, or PUFAs, which constitute the first line of defense against ROS-induced cell damage, including both enzymatic and non-enzymatic mechanisms [54,55]. Since *S. salina* cells can be subjected to various conditions in terms of temperature, pH, and [NaCl], it was decided that we would explore the radical scavenging capacity of lipophilic and hydrophilic antioxidants, expressed in Trolox maximum productivity equivalents, in both ethanolic and water extracts, under every experimental condition.

Regarding AOX-EtOH extracts, the productivity of lipophilic antioxidant compounds is suggested to be favored under low temperatures, combined with an alkaline pH (≈9.5) and intermediate [NaCl] ≈ 16 g·L^−1^. An increase of 6% in AOX-EtOH productivity was estimated when compared to the mid-point condition. Several studies showed that a shift in abiotic factors such as light, temperature, salinity, or pH favor the accumulation of lipophilic antioxidants, in particular carotenoids, certain polyphenols, and PUFAs [41,56,57,58,59]. High temperatures are likely to induce the synthesis of AOX, but low temperatures can also trigger other mechanisms in the production of antioxidants, as was the case with *Synechocystis* [60]. In the case of *S. salina*, it appears that the potential predictive response has to cope with low temperatures. Low temperatures and high salinities were indeed reported to enhance the PUFA content [8,56], as those fatty acids promote the fluidity of cell membranes, thus preserving the photosynthetic machinery and achieving physiological homeostasis [61,62].

On the other hand, the best conditions to achieve maximum productivity in terms of AOX compounds present in water extracts were not so different from that obtained with the control (≈20 °C, pH = 8.2, and [NaCl] ≈ 25 g·L^−1^). It is possible that these extracts, richer in PBPs, possess other types of antioxidant-related proteins and phenolic compounds (i.e., peptides) that are prone to occur under mild conditions (e.g., pH = 8).

### 4.5. Box-Behnken Design as an Optimization Tool for Cyanobacterial Production

Given the expected demand for more natural and healthier ingredients from cyanobacteria in the future, the development of novel and cost-effective technologies is a must, especially when novel strains of cyanobacteria have been unveiled. A major step towards that goal is to understand how environmental cultivation factors influence the production of cyanobacterial biomass and their bioactive pigments and antioxidants in food and nutraceutical industries as part of an overall optimization strategy.

The use of statistical factorial optimization, as is the case of a Box–Behnken design (among others, such as a central composite design), as a tool can help pinpoint the interaction effects between several abiotic parameters as well as identify their individual influence. This is considered a reliable statistical tool which has already been employed in many cyanobacterial and microalgal biomass and metabolite optimization-related studies [21,63,64,65,66]. From a reduced number of experimental runs, it allows us to determine which are the most relevant factors at stake and, subsequently, the best conditions to maximize productivity in terms of biomass and bioactive compounds of interest. This contrasts with the one-factor-at-a-time approach followed by conventional optimization studies, which can be limiting, more time-consuming, and unable to detect interactive synergistic or antagonistic effects between factors or offer a satisfying level of reliability [67].

A Box–Behnken factorial design was therefore employed to ascertain the interaction effects between temperature, pH, and [NaCl] on the productivities of biomass, pigments, and antioxidant compounds, as found in ethanolic and aqueous extracts. It confirmed that the three factors influence the metabolism of *S. salina* in different ways. In terms of biomass, [NaCl] appears to have a higher preponderance than other factors. Regarding pigments and antioxidants, the influence of different factors was disparate. It would appear that lower salinity (10 g·L^−1^) and alkaline pH are relevant when maximizing pigment productivities (except PE), but both temperature and pH, ranging from 23–25 °C and 8–9.5, respectively, differ for carotenoids, total PBPs, and each PBP individually. The pigment that stood out was PE, for which the best productivity conditions are influenced by a low temperature (15 °C) and pH (6.5) under a higher degree of salinity (up to ≈26 g·L^−1^). On the other hand, lower temperatures (15–19 °C) and higher salinities (16–25 g·L^−1^) influence the AOX compound productivities in both ethanolic and aqueous extracts. However, lipophilic compounds are influenced by a higher pH (≈9.5), while aqueous antioxidants are favored by an intermediate pH (=8).

It is worth mentioning that the optimal conditions for metabolite productivity may not coincide with their maximum content in the cyanobacterium cell; however, the accumulation of the final product within a short period of time is relevant in the context of bioprocess decision-making over a high content of pigment because this can imply longer cultivation with larger process-associated costs.

## 5. Conclusions

Overall, the Box–Behnken model predicted the best conditions to enhance biomass, pigment production, and antioxidant capacity productivities of strain *S. salina*. The model could predict a 2.7-fold increase in biomass productivity under optimized conditions, whilst for pigments, PE stood out, with a 2.3-fold increase, as did carotenoids and total PBPs, with 1.90- and 1.39-fold increases, respectively. For lipophilic antioxidants, a 1.1-fold increase was found when compared to the non-optimized conditions; only for AOX-water compounds did the prediction entail a marginal improvement of 1.38%.

Nevertheless, further fundamental studies are still needed to complement this information since other abiotic factors, such as light, nutrient availability, or even biotic factors (e.g., the presence of bacteria), are relevant to the overall effective cost of upstream processing. An ideal, large-scale scenario of a biorefinery process, in which biomass use is maximized and associated with the co-production of several value added-compounds in a single batch, could be an option to alleviate some of the costs associated with the overall cyanobacterial bioprocess. This would, accordingly, aid competitiveness in the nutraceutical and food markets.

## Figures and Tables

**Table 1:** Experimental factorial design with temperature, pH, and [NaCl] for processing conditions of *S. salina* (*n* = 3).

	Experimental Factors		
Runs	A: Temperature (°C)	B: pH	C: [NaCl] (g·L^−1^)
1	15	6.5	25
2	15	8	10
3	15	8	40
4	15	9.5	25
5	20	6.5	10
6	20	6.5	40
7	20	8	25
8	20	9.5	10
9	20	9.5	40
10	25	6.5	25
11	25	8	10
12	25	8	40
13	25	9.5	25

**Table 2:** Statistical significance of Box–Behnken model for all parameters and corresponding equations for the predicted response.

Parameters (Objective Function)	*p*-Value	R^2^	Equation
P_x_ (mg·L^−1^·d^−1^)	<0.0001	0.98	232.67 + 8.17 T − 10.75 pH − 141.75 [NaCl] + 18.75 T × pH − 16.08 T × [NaCl] + 22.42 pH × [NaCl] + 87.21 T^2^ − 23.79 pH^2^ + 149.37 [NaCl]^2^
Total carotenoids (mg·L^−1^·d^−1^)	<0.0001	0.72	0.1690 + 0.0386 T + 0.0159 pH − 0.0219 [NaCl] − 0.0165 T × pH − 0.0045 T × [NaCl] − 0.0020 pH × [NaCl] + 0.0288 T^2^ − 0.0423 pH^2^ + 0.0647 [NaCl] ^2^
T_PBP_ (mg·L^−1^·d^−1^)	<0.0001	0.81	2.22 + 0.2687 T− 0.0358 pH − 0.2182[NaCl] + 0.4686 T × pH − 0.0596 T × [NaCl] − 0.3596 pH × [NaCl] − 0.6547 T^2^ + 0.1737 pH^2^ − 0.0484 [NaCl] ^2^
PC (mg·L^−1^·d^−1^)	<0.0001	0.86	1.66 + 0.340 T + 0.1933 pH − 0.2354 [NaCl] + 0.1055 T × pH − 0.1265 T × [NaCl] − 0.0334 pH × [NaCl] − 0.4497 T^2^ − 0.2247 pH^2^ − 0.1265 [NaCl]^2^
APC (mg·L^−1^·d^−1^)	<0.0001	0.85	0.7785 + 0.0121 T − 0.0330 pH − 0.2216 [NaCl] + 0.2486 T × pH − 0.0439 T × [NaCl] − 0.0526 pH × [NaCl] − 0.1351 T^2^ − 0.0409 pH^2^ − 0.1403 [NaCl]^2^
PE (mg·L^−1^·d^−1^)	<0.0001	0.91	0.4306 − 0.0047 T − 0.0512 pH—0.0317 [NaCl] + 0.3337 T × pH + 0.0126 T × [NaCl] − 0.0783 pH × [NaCl] − 0.0222 T^2^ + 0.1471 pH^2^ − 0.1927 [NaCl]^2^
AOX-EtOH (mg_TE_·L^−1^·d^−1^)	<0.0001	0.92	1.53 − 0.0445 T + 0.4679 pH − 0.1503 [NaCl] − 0.3758 T × pH − 0.0756 T × [NaCl] + 0.1469 pH × [NaCl] + 0.1510 T^2^ − 0.5047 pH^2^ + 0.1395 [NaCl]^2^
AOX-Water (mg_TE_·L^−1^·d^−1^)	<0.0001	0.90	1.26 − 0.1356 T + 0.1022 pH − 0.0120 [NaCl] − 0.0403 T × pH − 0.0800 T × [NaCl] − 0.0392 pH × [NaCl] − 0.3917 T^2^ − 0.2588 pH^2^ − 0.1617 [NaCl]^2^

P_x_—maximum biomass productivity; T_PBP_—total phycobiliproteins; PC—phycocyanin; APC—allophycocyanin; PE—phycoerythrin; AOX- EtOH—antioxidant compounds of ethanolic extract; AOX-Water—antioxidant compounds of water extract.

**Table 3:** Best conditions in terms of temperature (T), pH, and [NaCl] predicted by the model for each objective function, with maximum estimated productivities and corresponding desirability, and a comparison with reference experimental values observed at the central point (i.e., non-optimized conditions: T = 20 °C, pH = 8, [NaCl] = 25 g·L^−1^) and corresponding extent of increase as predicted by the model.

		Model Prediction					
Objective Function	Experimental Value of the Central Point (Control)	T (°C)	pH	[NaCl] (g·L^−1^)	Maximum Productivity	Desirability	Extent of Increase (%)
P_x_ (mg·L^−1^·d^−1^)	233.10 ± 4.01	25	7.50	10	637.43 ± 20.56	0.974	175%
Total carotenoids mg·L^−1^·d^−1^	0.172 ± 0.00	25	8.00	10	0.33 ± 0.04	0.992	91%
T_PBP_ (mg·L^−1^·d^−1^)	2.22 ± 0.06	22.9	9.48	10.9	3.09 ± 0.32	1	39%
PC (mg·L^−1^·d^−1^)	1.66 ± 0.04	22.9	8.96	10	1.97 ± 0.20	0.974	19%
APC (mg·L^−1^·d^−1^)	0.78 ± 0.02	25	9.50	10	1.17 ± 0.12	0.940	50%
PE (mg·L^−1^·d^−1^)	0.43 ± 0.03	15	6.50	26.3	0.99 ± 0.09	0.968	130%
AOX-EtOH (mg_TE_·L^−1^·d^−1^)	2.10 ± 0.01	15	9.45	16.3	2.24 ± 0.18	1	6%
AOX-Water (mg_TE_·L^−1^·d^−1^)	1.45 ± 0.02	19.2	8.20	25.5	1.47 ± 0.09	0.988	1.38%

**Table 4:** Identification of HPLC-PDA pigments (Chl *a* and carotenoids) of acetonic extracts of *S. salina* LEGE 06155 and corresponding retention times and contents (mean ± SD (*n* = 3)) expressed in µg·g_DW_^−1^. Different small case letters (a, b and c) mean *p* < 0.05 (one-way ANOVA, Tukey′s multiple comparisons test).

Peak	Retention Time (min)	Identified Pigment	Concentration of Pigment (µg·g_DW_^−1^)
1	10.2	Unidentified carotenoid *	2.18 ± 0.18 ^a^
2	13.2	Zeaxanthin	3.26 ± 0.12 ^a^
4	24.6	Chl *a*	63.25 ± 1.16 ^b^
5	25.2	Echinenone	0.35 ± 0.01 ^c^
6	25.8	Unidentified carotenoid *	0.68 ± 0.04 ^c^
7	32.8	β-carotene	3.41 ± 0.07 ^a^

* Concentration expressed in an internal standard (trans-β-Apo-8′-carotenal) equivalents.

## Data Availability

The authors confirm that the data supporting the findings of this study are available within the article.
